# Quercetin targets SarA of methicillin-resistant *Staphylococcus aureus* to mitigate biofilm formation

**DOI:** 10.1128/spectrum.02722-23

**Published:** 2023-11-29

**Authors:** Panpan Liu, Xinyun Kang, Xiaohui Chen, Xiaofeng Luo, Caixia Li, Guiqin Wang

**Affiliations:** 1 College of Animal Science and Technology, Ningxia University, Yinchuan, China; The Pennsylvania State University, University Park, Pennsylvania, USA

**Keywords:** anti-biofilm, virtual screening, natural drugs

## Abstract

The emergence of methicillin-resistant *Staphylococcus aureus* (MRSA) led to the failure of conventional antibiotic treatment, resulting in serious morbidity and mortality. The biofilm formation of *S. aureus* is a potential mechanism for drug resistance. SarA is an important regulatory protein for the formation of *S. aureus* biofilm. In this study, the global regulator SarA was used as a target to virtually screen natural active monomers. SarA inhibitor quercetin (QEN) was assessed. Molecular docking and kinetic simulation showed that QEN was stably bound to the formation site of SarA dimer. Spectral analysis verified that QEN was bound to SarA and changed its conformation. *In vitro* biofilm, the culture showed that 4 µg/mL QEN significantly inhibited biofilm formation, and the production of extracellular polymers and eDNA concentration significantly reduced. Scanning electron microscopy and laser confocal microscopy further confirmed the inhibitory effect of QEN on biofilm formation. At the same time, a decrease in the transcription level of *sarA* and its downstream genes related to biofilm regulation and a decrease in the expression level of SarA were also observed, confirming the QEN-induced inhibition of *sarA*. In conclusion, the results of this study revealed SarA-mediated anti-biofilm effect of QEN on MRSA.

**IMPORTANCE:**

Anti-biofilm is an important strategy against *Staphylococcus aureus* chronic infection. SarA is a positive regulator of biofilm formation in *S. aureus*. In this study, we identified the SarA inhibitor quercetin using computer simulation screening. Previous studies have shown that quercetin inhibits biofilm; however, the underlying mechanism remains unknown. This study revealed the inhibitory effect of quercetin on the SarA protein. We also isolated the SarA protein and confirmed its interaction with quercetin *in vitro*. Besides, the inhibitory effect of quercetin on the transcription and translation levels of the SarA protein was also determined. The effects of quercetin on *S. aureus* biofilm inhibition and biofilm components were consistent with the changes in the transcription level of biofilm-related genes regulated by SarA. In summary, our study revealed the mechanism by which quercetin affects biofilm formation by inhibiting the transcriptional regulator SarA of *S. aureus*.

## INTRODUCTION

*Staphylococcus aureus* is one of the important pathogens in humans and animals. World Health Organization has recognized methicillin-resistant *Staphylococcus aureus* (MRSA) as an important *S. aureus* strain and multidrug-resistant (MDR) pathogen. *S. aureus* biofilm is an important cause of chronic infection and medical device contamination (1, 2). Removing mature biofilm is challenging, and biofilm bacteria have higher antibiotic resistance than planktonic bacteria (3). Biofilms resist antibiotics through multiple mechanisms, such as antibiotic neutralization, altered antibiotic permeability, growth metabolism, and high gene transfer rates (4, 5). Thus, anti-biofilm is an important strategy against *S. aureus* chronic infection.

*S. aureus* biofilm formation could be categorized as ica-dependent and ica-independent pathways. Polysaccharide intracellular adhesin (PIA) is the primary extracellular polymer in *S. aureus* biofilm, which is produced by the intracellular adhesin (icaABCD) operon. It mediates bacterial adhesion and reproduction during biofilm formation and provides structural integrity to the biofilm (6). SarA can bind to the promoter of the ica operon to promote its expression (7). Most *S. aureus* carry the ica operon, but are strain- and environment-dependent (8). Previous studies have shown that biofilm formation was unaffected in UASM-1 ica knockouts but SarA mutants showed weakened biofilm formation (9). Mutant MRSA SarA failed to form a biofilm, whereas MRSA strains formed biofilms predominantly in an ica-independent pathway (10).

SarA is a positive regulator of biofilm formation in *S. aureus*, and its partial mechanism is to negatively regulate protease and nuclease activities (11, 12). In the SarA-regulated biofilm formation, the extracellular protease-mediated mechanism is more important than the PIA-dependent mechanism, and in this process, SarA was not dependent on the agr pathway (13). The ica-independent pathway mainly involves proteins and eDNA. Extracellular proteases play a key role in protein-dependent biofilm formation. SarA can inhibit the activity of extracellular proteases (14). Mutations in SarA can lead to increased secretion of extracellular proteases including aureolysin (Aur), serine proteases (SspA and SplA-F), and cysteine proteases (ScpA/SspB), limiting biofilm formation of which Aur showed the highest impact on biofilm formation (15, 16). eDNA plays an important role in the early adhesion and maturation of ica independent *S. aureus* biofilm formation. The level of eDNA in biofilm is regulated by thermostable nuclease (Nuc), and multiple studies have shown that SarA could down-regulate the Nuc expression (17, 18).

**Fig 1:**
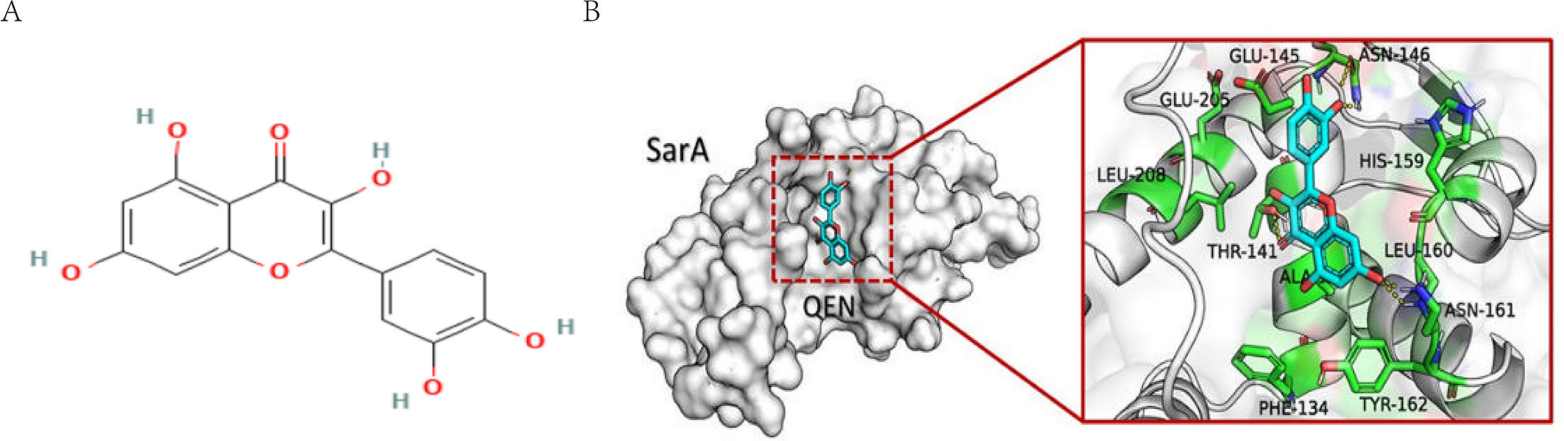
Analysis of interaction sites between QEN and SarA. (**A**) Structural formula of QEN; (**B**) interaction site between QEN and the SarA protein.

In this study, natural small-molecule QEN was screened by virtual screening of inhibitors targeting SarA. Quercetin (3,5,7,3ʹ,4ʹ-pentahydroxy flavone, QEN) belongs to the flavonol subclass of flavonoids. QEN is widely distributed in vegetables and fruits, including medicinal plants such as *Hypericum perforatum,* also known as Ginkgo. The molecular formula of QEN is C_15_H_10_O_7_, and its chemical structure is depicted in Fig. 1A. Its biological activity is mainly derived from its phenolic hydroxyl group and a double bond (19). QEN possesses antioxidant, anti-inflammatory, antiviral, anticancer, antibacterial, and neuroprotective characteristics. Besides, it also serves as a cardiovascular disease drug (20). Previous studies on the pharmaceutical properties of QEN showed that it acts as a natural antibacterial agent against many pathogenic microorganisms (21). In recent years, active compounds isolated from different fruits, vegetables, and other plants have attracted wide attention due to their beneficial effects on human health. QEN has been recognized as GRAS (Generally Recognized As Safe) by the United States Food and Drug Organization (22).

Previous studies have reported that QEN has an inhibitory effect on *S. aureus* biofilm. Computational simulations have suggested a potential mechanism by which QEN may bind to the *S. aureus* biofilm formation-related protein ClfB and thus influence bacterial colonization and adhesion to inhibit biofilm formation (23). However, the exact mechanism remains unclear. SarA is a positive regulator of biofilm formation in *S. aureus*. In this study, the global regulator SarA was used as a target to virtually screen natural active monomers. According to molecular docking and dynamics simulation, a stable and strong interaction exists between QEN and SarA. We achieved expression and purification of SarA and analyzed the fluorescent and infrared spectra of the QEN and SarA interaction, and it confirmed the interaction. In the *in vitro* biofilm culture, QEN inhibited the production of extracellular polymers and eDNA. Accordingly, transcription level analysis showed that QEN could down-regulate SarA and up-regulate the transcription levels of *aur* and *nuc* in the ica-independent pathway, and the decrease of SarA expression was verified at the protein level. The above results showed that QEN inhibited the biofilm formation of *S. aureus* by down-regulating the SarA expression and affecting the protein conformation by binding to SarA.

## RESULTS

### Molecular docking and dynamic simulation analysis

According to the screening results, we obtained 10 compounds with good docking scores (Table 1) and performed molecular docking and kinetic simulation analysis on quercetin, which has been reported to have anti-biofilm effects.

**TABLE 1:** Scores of the top 10 drugs acquired from screening

Name	Weight	Score	logP(o/w)
Myricetin	318.237	8.9286957	1.7589999
Trans-Zeatin-riboside	351.36301	8.521862	2.00594
Quercetin	302.23801	7.7761207	2.0320001
Oglufanide	333.34399	7.7615685	0.52499998
Camptothecin	348.358	7.6831865	2.3039999
Fisetin	286.23898	7.2354307	2.3050001
L-Abrine	218.256	7.0997972	1.767
Brevianamide F	283.33099	7.326756	1.405
Melatonin	232.28299	7.0609736	1.778
Dihydromyricetin	320.25299	7.0479846	1.173

The molecular docking results showed that the QEN was bound to the SarA protein at amino acids such as THR141, GLU145, ASN146, and ASN161 in the region to form six hydrogen bonds (Fig. 1). At the same time, the binding free energy reflects the obscurity in the interaction between QEN and SarA. The scoring function showed that QEN could bind to SarA protein with a free energy of −8.7 kcal/mol and form intermolecular forces such as Pi-sigma, hydrophobic bond, and van der Waals force, except for the hydrogen bond.

**Fig 2:**
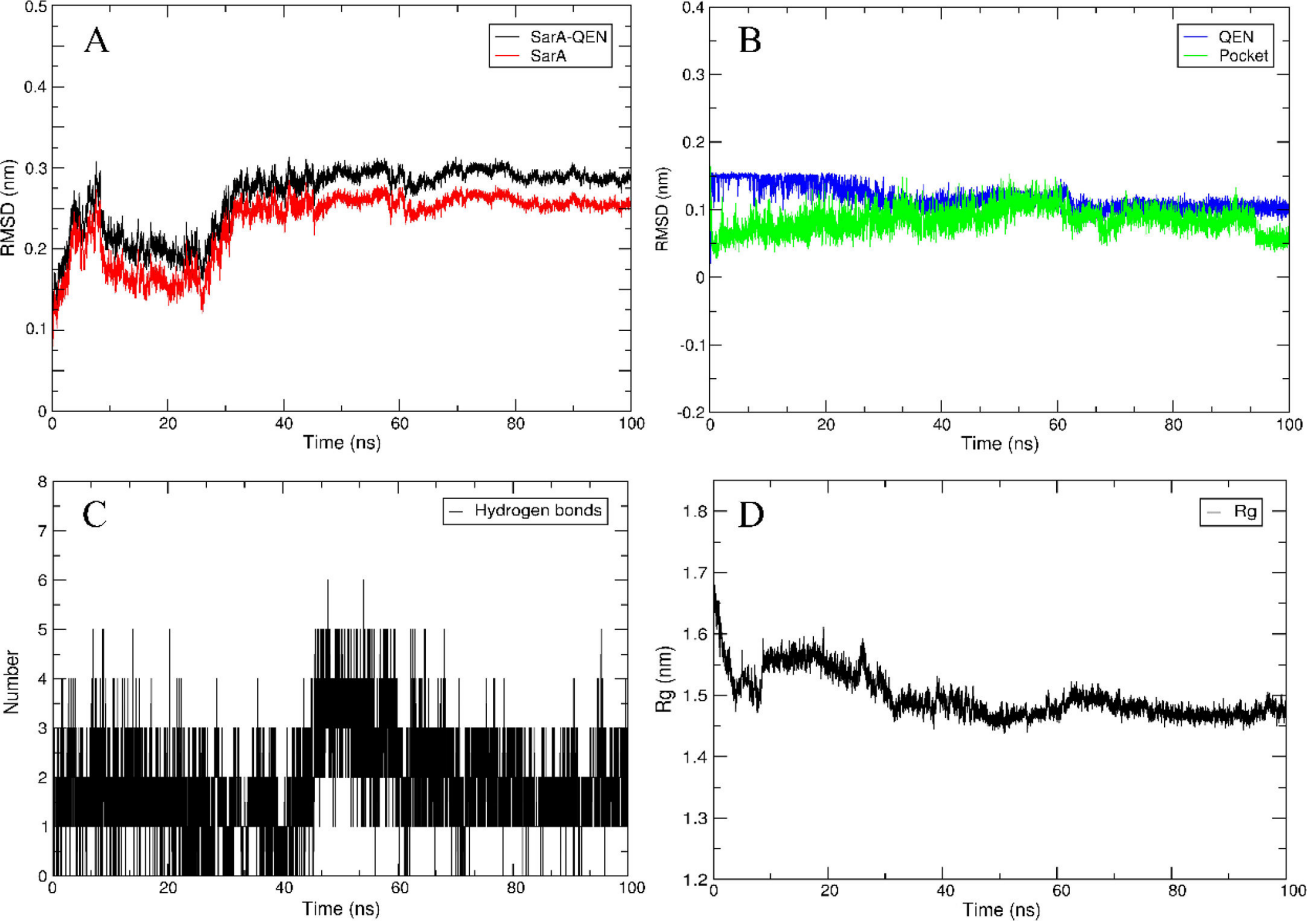
Dynamic simulation analysis of QEN and SarA. (**A**) RMSD value of the SarA-QEN complex; (**B**) RMSD value of the QEN-Pocket; (**C**) hydrogen bond network diagram; (**D**) radius of gyration of the SarA protein.

To further analyze the conformational changes in the SarA protein before and after binding to QEN and the stability of the resulting complex, the SarA-QEN complex was subjected to an all-atom dynamic simulation for up to 100 ns. The outcome of this analysis showed that the root mean square deviation (RMSD) value of the SarA-QEN complex (mean = 0.27 ± 0.08 nm) was slightly higher than that of the SarA protein (mean = 0.23 ± 0.04 nm). The overall structure of the complex tended to be stable and sustainable after 30 ns (Fig. 2A). At the same time, the QEN appeared to be more stable in the simulation process (mean = 0.13 ± 0.02 nm), and a significant change in conformation after 60 ns was not observed. No significant change was observed in the binding site of the amino acid (pocket) (mean = 0.12 ± 0.03 nm) (Fig. 2B). The conformation of QEN, SarA, and the QEN-SarA complex was found to be stable. In addition, QEN can stably form 3–4 hydrogen bonds with SarA during the whole simulation process (Fig. 2C). The radius of gyration of the SarA protein decreased and tended to converge after 30 ns, and then it slightly fluctuated by 1.5 nm (Fig. 2D). These results indicate that the binding modes of QEN and SarA proteins have certain reliability and stability.

### QEN inhibits *S. aureus* biofilm formation

**Fig 3:**
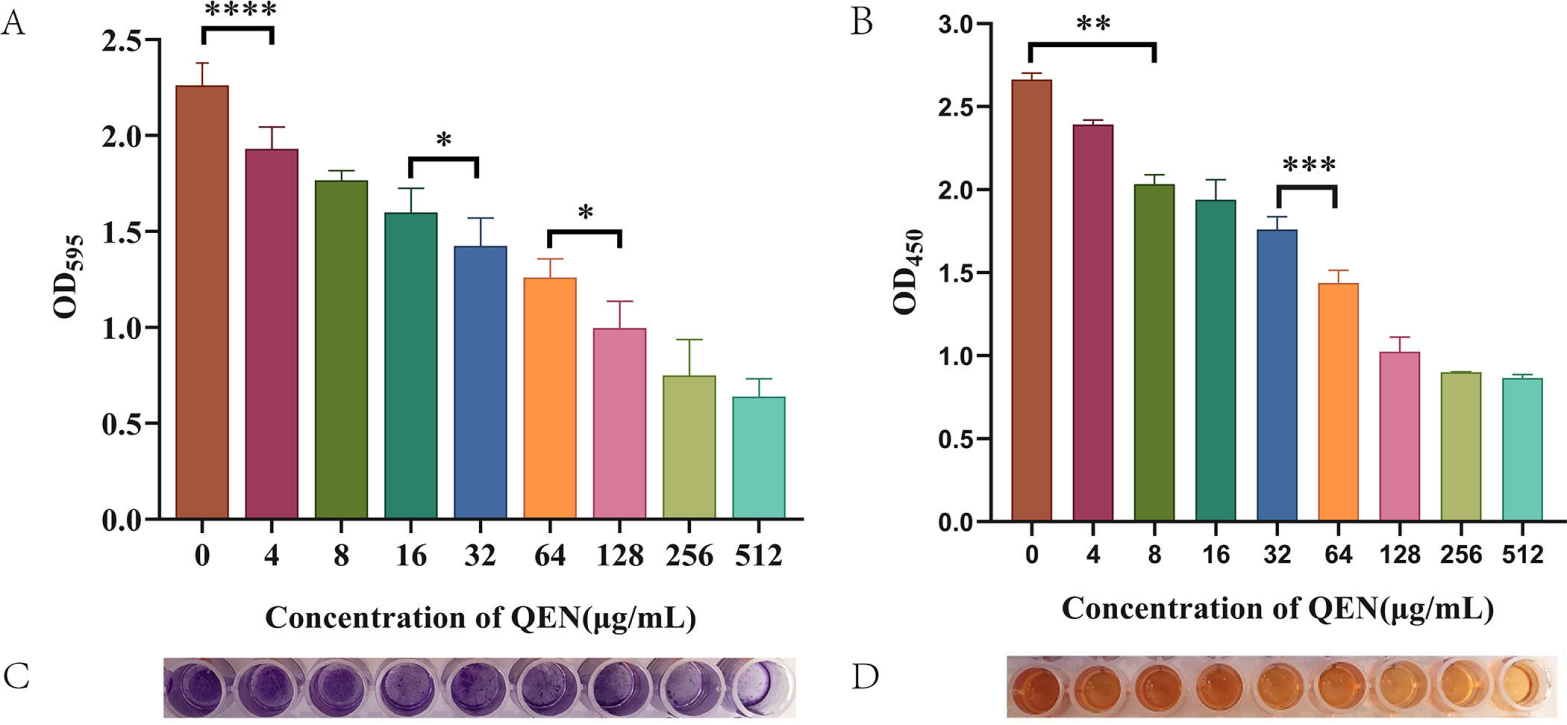
Effect of QEN on *S. aureus* biofilm formation. (**A**) Crystal violet quantification of biofilm; (**B**) 2,3- bis(2-methoxy-4-nitro-5-sulfophenyl)-5-[(phenylamino)carbonyl]-2H-tetrazolium hydroxide (XTT) quantification of biofilm; (**C**) crystal violet staining of biofilm; (**D**) XTT coloration. **** means *P* < 0.0001, *** means *P* < 0.001, ** means *P* < 0.01, * means *P* < 0.05, and ns means *P* > 0.05.

The effect of different concentrations of QEN on biofilm formation was determined using crystal violet. As shown in Fig. 3A and C, with increasing concentrations of QEN, the biofilm formation of *S. aureus* decreased gradually. QEN showed a concentration-dependent inhibitory effect on the biofilm formation of *S. aureus* MRSA 33591. After the QEN concentration increased to 128 µg/mL, the inhibitory effect gradually declined with no significant difference in biofilm formation.

### QEN inhibits the metabolic activity of biofilm bacteria

XTT is a mitochondrial dehydrogenase substrate that could be reduced by living cells into a water-soluble orange-yellow formazan dye. The absorption peak at 450 nm is proportional to the number of living cells. The number of viable cells in the biofilm was linearly correlated to the QEN concentration in line with the thickness of the biofilm. QEN concentrations >128 µg/mL also showed an inhibitory effect, but no significant difference was observed in biofilm inhibition (Fig. 3B and D).

### QEN inhibits the production of extracellular polymeric substances (EPS)

**Fig 4:**
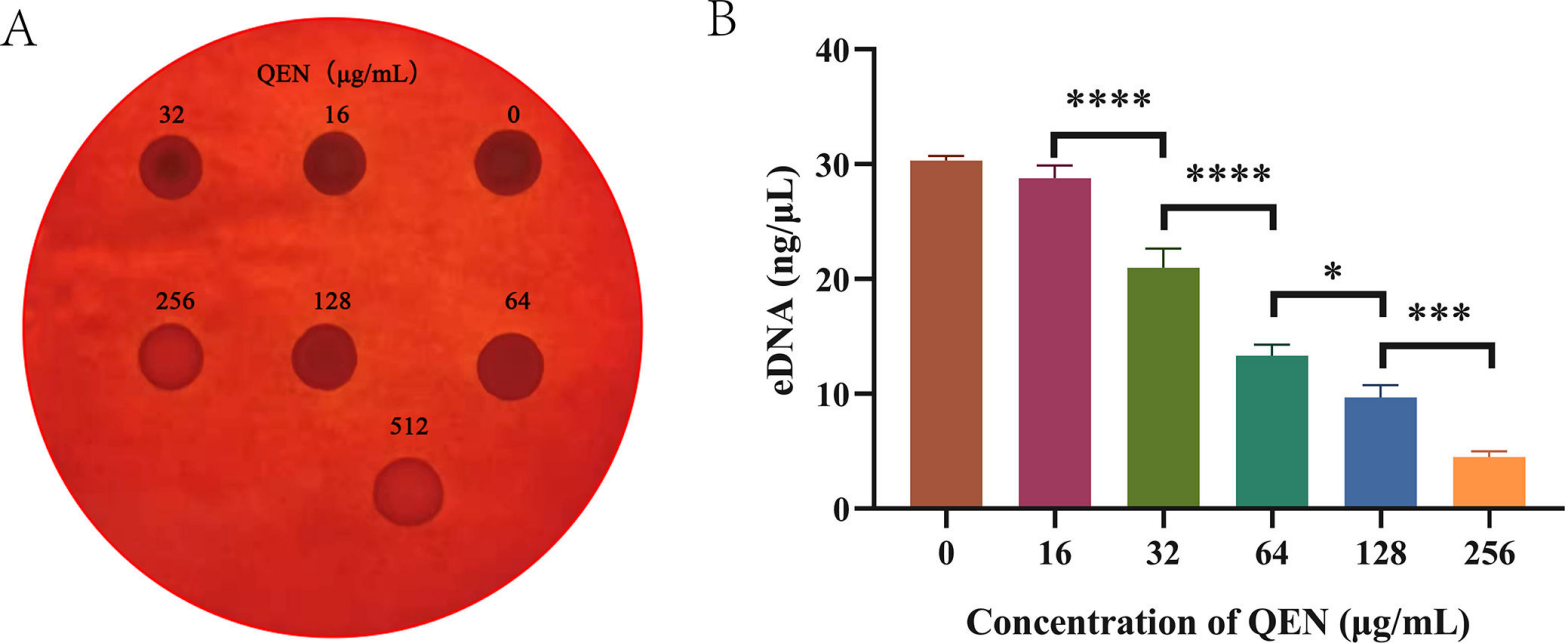
Effects of different concentrations of QEN on biofilm EPS. (**A**) Effect of QEN on biofilm EPS synthesis of MRSA 33591; (**B**) effect of QEN on eDNA synthesis of MRSA 33591. **** means *P* < 0.0001, *** means *P* < 0.001, ** means *P* < 0.01, * means *P* < 0.05, and ns means *P* > 0.05.

EPS is the main polysaccharide component in the *S. aureus* biofilm matrix and has a structural function. *S. aureus* was co-cultured using different concentrations of QEN on a Congo red plate (Fig. 4A). Compared with the control, the coloration gradually decreased with increasing QEN concentration, indicating that EPS synthesis decreased.

### QEN inhibits the eDNA production of membrane bacteria

eDNA is another matrix component of biofilm, most of which comes from the DNA released by the autolysis of bacteria, also known as the “binder” of biofilm. In the biofilm cultured with different concentrations of QEN, the secretion of eDNA was significantly inhibited with increasing QEN concentrations (Fig. 4B).

### Visualization of biofilm

**Fig 5:**
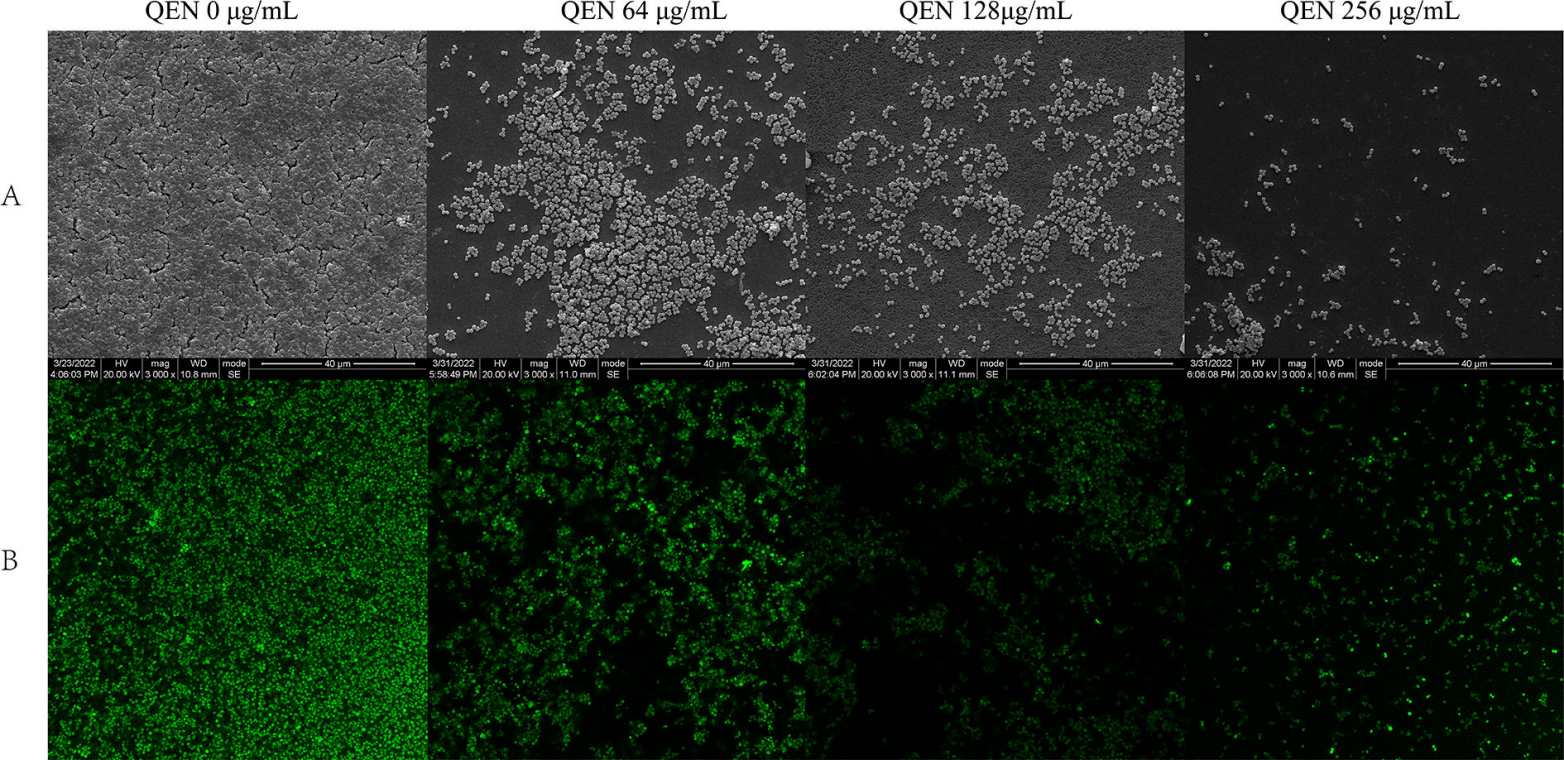
Microscopic observation of biofilm. (**A**) SEM of biofilm of MRSA 33591 (scale bar = 40 µm); (**B**) CLSM of biofilm of MRSA 33591 (63× oil lens).

The biofilms were cultured with QEN concentrations of 0, 64, 128, and 256 µg/mL. The effect of QEN on the morphology of the MRSA 33591 biofilm was observed using SEM. *S. aureus* formed a complete and dense biofilm wrapped in an extracellular matrix on the surface of the slide. At a QEN concentration of 64 µg/mL, the biofilm thickness and number of biofilm bacteria decreased significantly, the number of extracellular matrices decreased sharply, the biofilm bacteria showed a sheet distribution of small clusters, and the biofilm appeared as a single-layer cell aggregation. At 128 µg/mL, a sparse single or several bacteria adhered to the surface of the slide. At a QEN concentration of 256 µg/mL, only a single *S. aureus* cell was observed on the surface of the slide (Fig. 5A). The biofilm was stained with SYTO^TM^ 9/PI, and the survival of the biofilm bacteria was observed under a laser confocal microscope. No dead cells were observed in the biofilm cultured with different concentrations of QEN; however, some cells appeared yellowish-green (Fig. 5B).

### The effect of QEN on SarA-regulated membrane-associated genes

**Fig 6:**
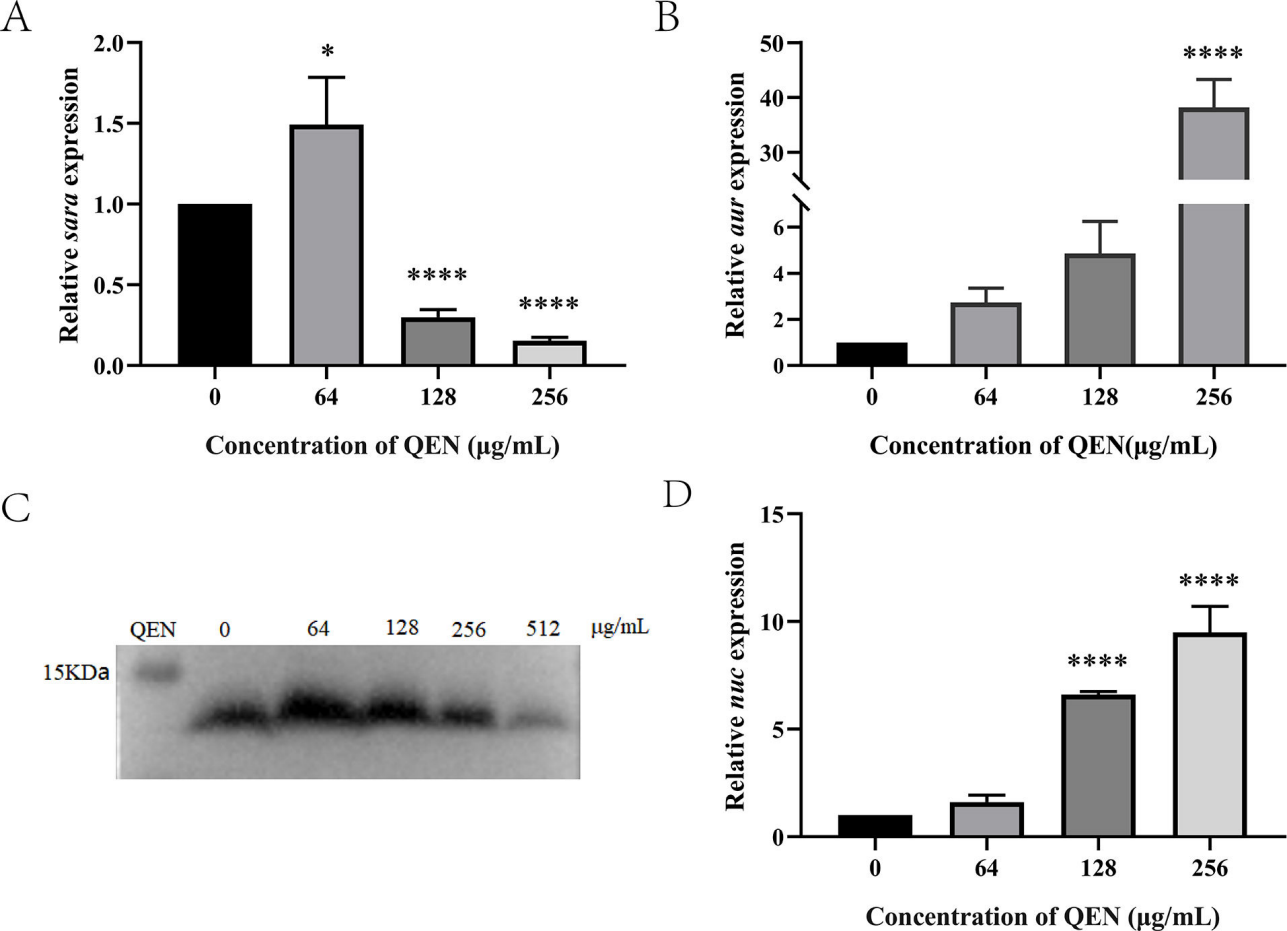
Effects of QEN on SarA and its downstream genes. (**A**, **B**, **D**) Effects of QEN on the transcriptional levels of *sarA*, *aur,* and *nuc*; (**C**) quercetin inhibited the expression of SarA.

The transcriptional level of biofilm-related genes regulated by SarA was analyzed using qPCR. The results showed that the transcription level of SarA decreased under different concentrations of QEN in a concentration-dependent manner. Transcriptional analysis of downstream membrane-associated genes negatively regulated by SarA showed that the transcription levels of extracellular metalloproteinase Aur (aureolysin, Aur) and extracellular nuclease Nuc (nuclease) were significantly increased (Fig. 6A, B, and D).

### The effect of QEN on the expression of the SarA protein in *S. aureus*

Western blot analysis of SarA protein in biofilm bacteria co-cultured with different concentrations of QEN was conducted. The results showed that QEN inhibited the expression of SarA in a dose-dependent manner (Fig. 6C).

### Interaction analysis between QEN and SarA *in vitro*

### Fluorescence spectrum

**Fig 7:**
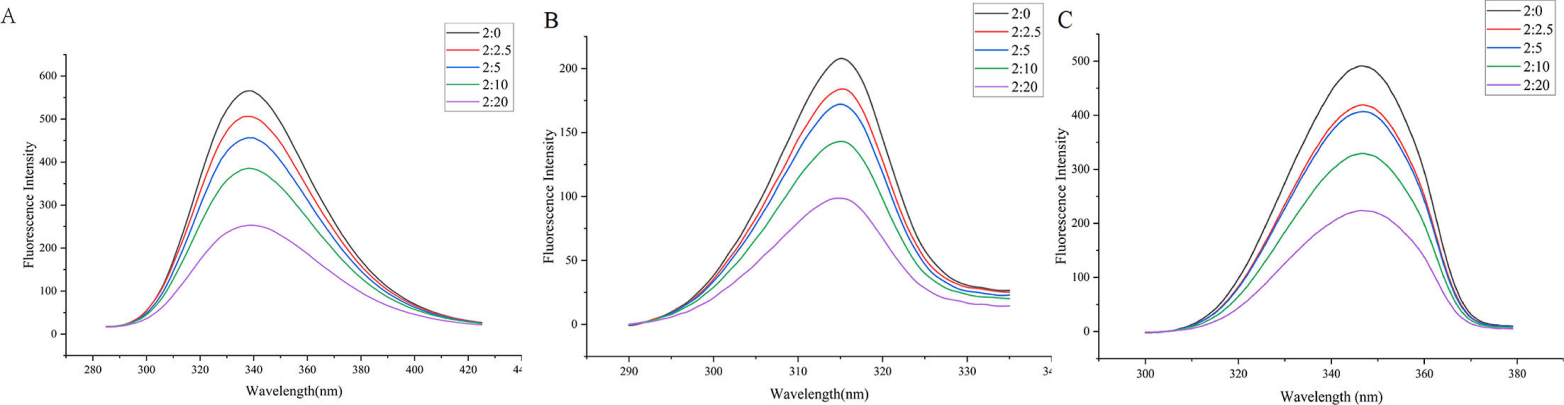
Effect of concentration of QEN on fluorescence intensity of SarA before and after addition of quercetin. (**A**) Fluorescence emission spectra of SarA in the presence of QEN; (**B**) synchronous fluorescence spectra of SarA in the presence of QEN (Δλ = 15 nm); (**C**) synchronous fluorescence spectra of SarA (2 µM) in the presence of QEN (Δλ = 60 nm). From top to bottom, the molar ratios of SarA:QEN are 2:0, 2:2.5, 2:5, 2:10, and 2:20, respectively.

Proteins can produce endogenous fluorescence under ultraviolet light excitation as they contain tryptophan (Trp) and tyrosine (Tyr). When small molecules bind to them and affect their conformation, the intensity of endogenous fluorescence decreases. The characteristic fluorescence information of tyrosine and tryptophan residues could be obtained at 15 and 60 nm, revealing the influence of small molecules on protein conformation. The synchronous fluorescence spectrum of tryptophan residues is usually used as a qualitative basis for judging the change in protein conformation (24, 25). Fluorescence spectra were measured after incubating with SarA:QEN at 2:0, 2:2.5, 2:5, 2:10, and 2:20 for 1 h at 37°C. The results showed that with an increasing QEN addition ratio, the fluorescence emission peak of SarA decreased and the fluorescence quenching effect on SarA increased (Fig. 7A). At Δλ = 15 nm and Δλ = 60 nm, the peaks of the synchronous scanning spectra gradually decreased, but the peak positions did not change. This indicated that QEN formed a complex compound with SarA but did not affect the microenvironment of tryptophan and tyrosine in SarA (Fig. 7B and C).

### Infrared spectrum

**Fig 8:**
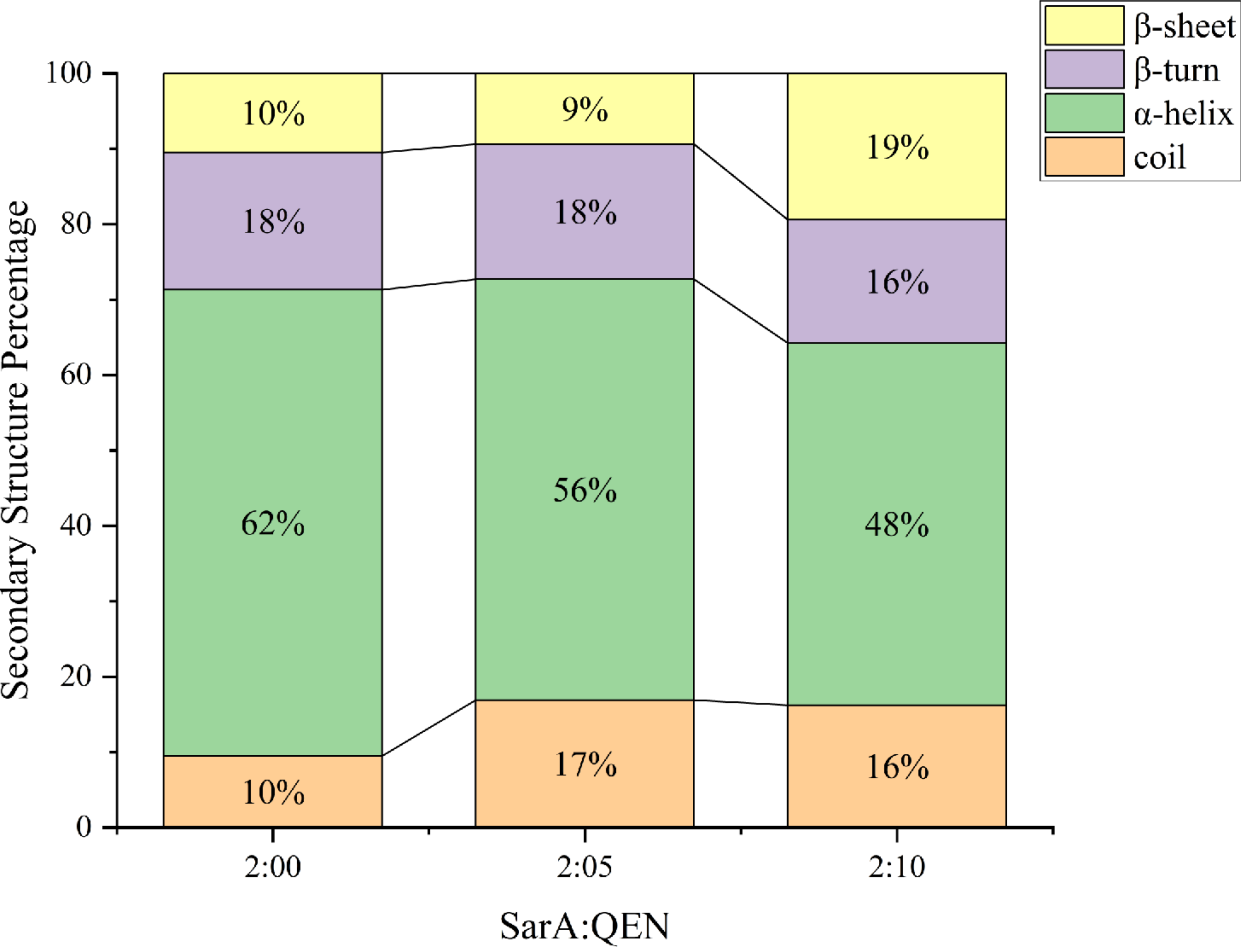
Effect of QEN on the secondary structure of SarA.

The secondary structure content of SarA at different incubation concentrations of QEN was calculated by selecting the infrared spectrum amide I band (1,700–1,600 cm^−1^). The results showed that with increasing QEN:SarA molar ratio, the α-helix content of the SarA protein gradually decreased and the random coil β-sheet content increased. It indicated that part of the α-helix of the SarA protein gradually changed into a random coil and part of the protein structure changed from an ordered structure to a disordered structure (Fig. 8).

## DISCUSSION

*S. aureus* forms biofilms to resist antibiotics and the host immune system. Thus, treatment of infections that are associated with biofilm formation on medical devices and implants, osteomyelitis infections in adults, and chronic wound infections is extremely challenging (5, 26 – 29). SarA has been identified as a target for the development of antibacterial drugs for *S. aureus* (30). In this study, the SarA inhibitor QEN was obtained by virtual screening. Molecular docking results showed that QEN binds to SarA and forms six hydrogen bonds to affect dimer formation. The hydrogen bond is an important force to stabilize the ligand-receptor complex, and the number of hydrogen bonds is positively correlated to the stability of the complex to a certain extent. The kinetic simulation showed that the conformations of the QEN-SarA complex were stable, indicating that the binding mode of QEN and SarA is reliable and stable.

SarA inhibitors are often reported to have anti-biofilm effects (31 – 33). The results of biofilm formation and metabolic activity of biofilm bacteria indicated the anti-biofilm effect of QEN. We observed that at a concentration of 4 µg/mL, QEN inhibited biofilm formation and growth of biofilm bacteria in a concentration-dependent manner. To observe the inhibitory effect of QEN on biofilm formation, SEM analysis was performed to unravel the effect of QEN on the morphological structure of *S. aureus* biofilm. *S. aureus* could form a complete and dense biofilm wrapped in an extracellular matrix on the surface of the slide. When the concentration of QEN was 64 µg/mL, biofilm formation and biofilm bacteria decreased significantly, the number of extracellular matrices decreased sharply, and the biofilm showed a sheet-like distribution of small clusters. At this time, the biofilm showed a single layer of cell aggregation, and at 128 µg/mL, a sparse single or several bacteria adhered to the surface of the slide. At 256 µg/mL, only a single *S. aureus* cell was observed on the surface of the slide.

*S. aureus* produces EPS, an extracellular matrix that promotes *S. aureus* adhesion and biofilm formation, increases its ability to colonize host tissues, enhances bacterial persistence, and confers resistance to the host immune response (34, 35). Congo red plate qualitative detection of MRSA EPS formation showed that with increasing QEN concentration, biofilm extracellular matrix synthesis decreased, indicating that QEN inhibited MRSA mucus production. As the main component of MRSA biofilm, eDNA-mediated intracellular adhesion and self-aggregation. The eDNA was extracted and detected. We observed that the eDNA content decreased with increasing QEN concentrations. The above results indicated that QEN inhibited the synthesis of EPS and eDNA in biofilm bacteria, affecting biofilm formation in *S. aureus*.

The ica-independent pathway of biofilm formation mainly involved proteins and eDNA. Extracellular proteases play an important role in the protein-dependent pathway. Increased secretion of extracellular proteases limits biofilm formation, and metalloproteinase Aur showed the highest inhibitory effect (16, 36). eDNA is abundant in *S. aureus* biofilm and is involved in improving the structural stability of biofilms, horizontal gene transfer, and the development of antibiotic resistance (37). The level of eDNA in the biofilm is regulated by thermostable nuclease (Nuc), which could degrade eDNA and reduce biofilm formation (18, 38, 39). A plethora of studies have shown that the transcription of *aur* and *nuc* is negatively regulated by SarA. In this study, QEN down-regulated the *sarA* transcription level in MRSA 33591, which is in line with the study by Lee et al. (40). In addition, the transcription of *aur* and *nuc* was up-regulated, indicating that QEN could inhibit biofilm formation by MRSA 33591 by inhibiting *sara* and downstream ica-independent pathways. Consistent with the results at the transcription level, western blot analysis of SarA showed that the expression of SarA in biofilm bacteria also decreased with an increasing concentration of QEN. The above results indicated that QEN has an anti-biofilm effect, which is conferred by inhibiting the transcription and expression of the SarA protein, thereby disrupting the regulation of downstream biofilm-related genes.

To validate the molecular docking results, we obtained high-purity SarA protein by prokaryotic expression and purification. The excitation wavelength of the SarA protein was 287 nm. The fluorescence spectra of QEN and SarA showed that the endogenous fluorescence quenching of SarA gradually increased with an increasing molar ratio of QEN to SarA. In the synchronous scanning fluorescence spectra of tyrosine (Tyr) with Δλ = 15 nm and tryptophan (Trp) with Δλ = 60 nm, the fluorescence intensity gradually decreased with the QEN addition, indicating that SarA interacted with QEN to form a complex. The secondary structure content of SarA was obtained by Fourier transform infrared spectroscopy. We observed that with increasing concentrations of QEN, the content of the α-helix of the SarA protein decreased and that of the random coil increased. This result is consistent with the molecular docking results, which prove that QEN interacts with SarA and that QEN interaction can alter SarA conformation.

### Conclusion

In this study, QEN was screened as a SarA inhibitor. QEN showed anti-biofilm effect on *S. aureus* at 4 µg/mL. We predicted the binding model of SarA and QEN and confirmed the interaction by spectral analysis. QEN interacts with SarA to promote the transformation of its α-helix structure into a random coil and change its conformation. Furthermore, molecular biology experiments showed that QEN inhibited transcription and decreased the expression of SarA. Consistent with this, the transcription levels of the downstream regulatory genes of SarA (*aur* and *nuc*) were up-regulated. In conclusion, QEN targets SarA to play an anti-biofilm role.

## MATERIALS AND METHODS

### Bacterial strain and growth condition

The standard MRSA strain (ATCC-33591) and the isolated strains used in this study were preserved in our laboratory. The culture condition of the biofilm was Trypticase soy broth (TSB) medium containing 0.5% glucose (TSB-g). *S. aureus* was cultured to the logarithmic growth phase, and the bacterial suspension was adjusted to an OD_600_ of 0.1 (about 1 × 10^8^ CFU/mL). After 100-fold dilution with TSB-g, QEN storage solution (40,960 µg/mL) was added to prepare a suspension of bacteria with different QEN concentrations. *E. coli* DH5α and BL21 (DE3) were purchased from Tsingke Biotechnology Co., Ltd.

### Virtual screening of SarA inhibitors

The SarA protein crystal structure (PDB ID: 2FRH) from *S. aureus* was obtained from the RCSB PDB database. The candidate drug molecules were obtained in batches from the drug databases, such as ZINC (http://zinc.docking.org/), PubChem (https://pubchem.ncbi.nlm.nih.gov/), Drug Bank (https://go.drugbank.com/), TCMSP (https://tcmspw.com/tcmsp.php), and Specs (https://www.specs.net/). A local database containing 2,400 natural active monomers was constructed, and several molecules were confirmed to have biofilm inhibitory activity. The interaction affinity between the target protein and some drug molecules was preliminarily analyzed using the Bing DB database. Subsequently, small molecules were docked with the SarA protein using molecular docking tools such as Auto Dock Vina, Q Vina 2, SMINA, and UCSF DOCK 6. The candidate molecules were subjected to energy minimization in the MMFF94 force field, followed by semi-flexible and flexible molecular docking analyses. The classical scoring function was used to evaluate the molecular docking results. The preferred indicators were the binding-free energy and the degree of conformational fit. The binding free energy was calculated using the formula: E_Interaction_ = E_Vdw_ + E_Electrostatic_ + E_H-bond_. The binding mode was analyzed and visualized using PyMOL 2.4, LigPlus 2.2, Discovery Studio 2019, and the online tool Proteins Plus.

### Prediction of the binding of SarA and QEN by molecular dynamics simulation

To analyze the existing mechanism of action and verify the reliability of the binding mode, the classical molecular dynamics simulation software GROMACS 2019.06 was used to perform an all-atom molecular dynamics simulation with the docked SarA-QEN complex as the initial conformation. Amber 99 SB-ILDN force field parameters were used for receptor proteins and QEN molecules. UNK molecular topology files were generated with the help of the ACPYPE Server (https://www.bio2byte.be/acpype/) and by selecting dodecahedron solvation. The nearest distance between the system boundary and the complex was set to 1.0 nm. The TIP3P water model was selected, and Na^+^ or Cl^–^ was randomly added to the complex system according to the VERLET cutting method to counterbalance the charge on proteins. Then, the system temperature was set to 300 K, and a constant pressure of 101.325 kPa was maintained by minimizing the system energy, NVT temperature control, and NPT pressure control. Based on the above balanced system, a free dynamics simulation of 100 ns was applied. The RMSD reflects the change in protein structure. The radius of gyration (Rg) reflects the degree of tightness before and after protein binding in the system. The number of hydrogen bonds reflects the stability of the complex.

### Effect of QEN on the biofilm formation ability of *S. aureus*

Previous reports have shown that the MIC of QEN is greater than 594 µg/mL (41), and our group also found that QEN of 512 µg/mL does not affect the growth of the planktonic state of *S. aureus* (42). Therefore, we determined the effect of 4–512 µg/mL QEN addition on *S. aureus* biofilm. Crystal violet is a basic protein dye that can stain negatively charged surface molecules and extracellular polysaccharide complexes. It can stain live bacteria, dead bacteria, and bacterial extracellular matrix and thus be used to quantify biofilm (43). The biofilm was cultured in a 96-well cell culture plate and incubated at 37°C for 40 h. The culture supernatant was discarded, and PBS was used to wash away planktonic bacteria. After complete drying, 200 µL 0.1% crystal violet staining was carried out for 15 min, and later the biofilms were washed with PBS for five times to remove excess crystal violet, dried, and later 200 µL of 95% ethanol was added to each well and oscillated for 10 min to release crystal violet from the biofilm. The absorbance at 595 nm (OD_595_) was measured.

### Determination of the metabolic activity of biofilm bacteria by XTT

XTT is a kind of tetrazolium salt as well as a substrate of mitochondrial dehydrogenase. It can be deacidized by enzymes in the cytoplasm of the respiratory chain to form a water-soluble formazan. The biofilm culture method was the same as the crystal violet method. The cells were washed three times with PBS and dried by inverting the 96-well plate. One hundred microliter of PBS and 100 µL of XTT-menadione solution were added to each pre-washed hole. Plates covered with aluminum foil were incubated at 37°C for 5 h. One hundred microliter of the solution was transferred to a new 96-well plate, and the absorbance was measured at 450 nm.

### Analysis of *S. aureus* biofilm morphology using SEM

The overnight culture of *S. aureus* 1:100 was diluted in TSB-g medium and cultured at 37°C for 40 h. The planktonic bacteria were washed with PBS, and the biofilm was fixed with 4% paraformaldehyde. The biofilm was later rinsed with ultrapure water for 5 min each time and dehydrated with a series of gradients of alcohol for 10 min each time. The sterile coverslip culture (1 cm × 1 cm) gently adhered to the conductive adhesive, ion sputtering spray, observed at 40 µm times.

### Observation of the survival subpopulation of *S. aureus* under the membrane by CLSM

After fixing the biofilm as mentioned above, biofilms were later fixed in 4% paraformaldehyde. PI (1 mg/mL) dye was used to stain the biofilm for 5 min in the dark and washed with PBS for five times, 3 min each time. The biofilms were later stained with SYTO 9 stain for 10 min and washed with PBS for five times. The working concentration of dye contained dye and PBS in a 1:100 ratio. These biofilms were later subjected to microscopic analysis (Leica Laser Confocal Microscopy, 10 × 63 oil lens).

### Determination of extracellular polymeric substances in biofilm

A Congo red plate binding assay was used to detect the synthesis of EPS in *S. aureus* biofilm treated with different concentrations of QEN. MRSA ATCC33591 was treated with QEN at concentrations of 16, 32, 64, 128, 256, and 512 µg/mL, respectively. Finally, 10 µL of the bacterial solution was added vertically to the Congo red plate and cultured in a 37°C incubator for 16 h. The effect of QEN on the synthesis of EPS was reflected in the color change.

### Determination of the eDNA of biofilm

The secretion of eDNA in *S. aureus* under different concentrations of QEN was detected using ultraviolet spectrophotometry. Two hundred microliter of *S. aureus* suspension (about 1 × 10^8^ CFU/mL) was added to the 24-well plate, and 2 mL of culture medium containing different concentrations of QEN was added to the 24-well plate to obtain a final concentration of 16, 32, 64, 128, and 256 µg/mL. At the same time, the medium without QEN was used as a blank control and cultured in an incubator at 37°C for 24 h. The eDNA was extracted by Trizol, and the OD_260_/OD_280_ ratio was detected using a spectrophotometer. The relative content of eDNA was expressed as eDNA content per unit biofilm (amount of biofilm in one hole of a 24-well plate) using three biological replicates.

### Quantitative real-time PCR analysis of biofilm-related genes

The *S. aureus* biofilm was cultured under the condition of QEN concentrations of 0, 64, 128, and 256 µg/mL. The Trizol method was used to extract the total RNA from the biofilm cultured in a 90-mm petri dish for 40 h, and qPCR was performed using a Bio-rad fluorescence quantitative PCR instrument after reverse transcription. The results were calculated using the 2^−ΔΔCt^ method (Table 2).

**TABLE 2:** Primers used in the real-time PCR analysis of biofilm-related genes

Primer	Sequence	Purpose
aur-F	CGCACATTCACAAGTTTATCGG	RT-PCR
aur-R	TTAGAGCGCCTGACTGGTCCTT	RT-PCR
nuc-F	ATCGCTTGCTATGATTGTGG	RT-PCR
nuc-R	TTCACCGTTTCTGGCGTAT	RT-PCR
q sarA-F	CAATTAGCTTTGAAGAATTCGCT	RT-PCR
q sarA-R	CGAAGTAATCTTCTTGAGATAAAAT	RT-PCR
gyrB-F	CGCAGGCGATTTTACCATTA	RT-PCR
gyrB-R	GCTTTCGCTAGATCAAAGTCG	RT-PCR
sarA-F	CccatggGCAATTACAAAAATCAATGATTG	Expression
sarA-R	CCctcgagTAGTTCAATTTCGTTGTTTGCTTC	Expression

### Expression, purification, and polyclonal antibody preparation of recombinant SarA

SarA was amplified from the genomic DNA of *S. aureus,* isolated, and inserted into the pET28A expression vector. The primer sequences of the target gene are listed in Table 1. The recombinant plasmid was transformed into BL21 (DE3) using the chemical transformation method. Prokaryotic expression of the SarA protein was induced by isopropyl β-D-thiogalactoside (IPTG) and purified using Ni-affinity purification. Protein concentration was determined using the bicinchoninic acid (BCA) method. The purified SarA was sent to the Chengdu Lilai Biomedicine Experiment Center to prepare a polyclonal antibody.

### Effect of QEN on SarA expression in *S. aureus*

The biofilm samples were prepared under the culture conditions of 0, 64, 128, and 256 µg/mL QEN and transferred to the polyvinylidene fluoride (PVDF) membrane after SDS-PAGE electrophoresis. The anti-SarA rabbit polyclonal antibody was used as the primary antibody, and the HRP-conjugated goat anti-rabbit IgG was used as the secondary antibody. The specific band of SarA was detected using ECL chemiluminescence.

### Fluorescence spectroscopy analysis of SarA and QEN *in vitro* binding

Fluorescence spectroscopy is the most commonly used method to study the interaction between small molecules and proteins. Fluorescence quenching refers to the process in which the interaction between the quencher molecule and the fluorescent group leads to a decrease in the fluorescence quantum yield of the latter through complex formation, energy transfer, collision quenching, and so on (44). SarA protein can produce endogenous fluorescence under ultraviolet light excitation because it contains tryptophan (Trp) and tyrosine (Tyr), which mainly show Trp characteristics. When the small molecule binds to it and affects the conformation, it will reduce the intensity of endogenous fluorescence. The more obvious the allosteric effect caused by the binding, the more the fluorescence intensity decreases. The fluorescence spectra were recorded using a 970 CRT fluorospectrophotometer with an excitation wavelength (λex) of 287 nm and a fluorescence emission wavelength (λem) range of 285–425 nm. The synchronous fluorescence spectra of SarA with and without QEN were scanned, recording Δλ = 15 nm (λem = 285–345) and Δλ = 60 nm (λem = 300–380). The scan speed was fixed at medium scanning speed.

### Infrared spectrum verification of the interaction between QEN and SarA *in vitro*

There are many characteristic absorption peaks in the infrared spectrum of protein, and the amide I band (1,700–1,600 cm^−1^) contains abundant secondary structure information, including α-helix, β-sheet, β-turn, random coil, and so on. Therefore, we detected the secondary structure changes of SarA before and after binding by infrared spectroscopy (45). QEN and SarA proteins were mixed in different molar ratios of 2:0, 2:5, 2:10, and incubated at 37°C for 1 h. SarA treated with different concentrations of QEN was scanned in the ATR attachment, and the water background was deducted before spectral scanning. The scan range was set to 4,000–400 cm^−1^ with a resolution of 4 cm^−1^. Infrared spectrum analysis was performed using Peak fit version 4.12 software in the spectral range (amide I band 1,700–1,600 cm^−1^) for baseline correction, smoothing, deconvolution, and Gaussian curve fitting based on the second derivative spectrum to determine the sub-peak. Corresponding protein secondary structure was predicted, and the peak area was obtained from secondary structure ratio.
